# Impaired uptake of conjugated bile acids and hepatitis b virus pres1-binding in na^+^-taurocholate cotransporting polypeptide knockout mice

**DOI:** 10.1002/hep.27694

**Published:** 2015-05-08

**Authors:** Davor Slijepcevic, Christina Kaufman, Catharina GK Wichers, Eduardo H Gilglioni, Florian A Lempp, Suzanne Duijst, Dirk R de Waart, Ronald PJ Oude Elferink, Walter Mier, Bruno Stieger, Ulrich Beuers, Stephan Urban, Stan FJ van de Graaf

**Affiliations:** 1 Tytgat Institute for Liver and Intestinal Research and Department of Gastroenterology and Hepatology, AMCAmsterdam, The Netherlands; 2 Department of Infectious Diseases and of Molecular Virology, University Hospital HeidelbergHeidelberg, Germany; 3 Department of Nuclear Medicine, University Hospital HeidelbergHeidelberg, Germany; 4 Department of Molecular Cancer Research, Section of Metabolic Diseases, University Medical Center UtrechtUtrecht, The Netherlands; 5 Department of Clinical Pharmacology and Toxicology, University Hospital ZurichZurich, Switzerland; 6 German Center for Infection Research, Heidelberg UniversityHeidelberg, Germany

## Abstract

The Na^+^-taurocholate cotransporting polypeptide (NTCP) mediates uptake of conjugated bile acids (BAs) and is localized at the basolateral membrane of hepatocytes. It has recently been recognized as the receptor mediating hepatocyte-specific entry of hepatitis B virus and hepatitis delta virus. Myrcludex B, a peptide inhibitor of hepatitis B virus entry, is assumed to specifically target NTCP. Here, we investigated BA transport and Myrcludex B binding in the first *Slc10a1*-knockout mouse model (*Slc10a1* encodes NTCP). Primary *Slc10a1^−/−^* hepatocytes showed absence of sodium-dependent taurocholic acid uptake, whereas sodium-independent taurocholic acid uptake was unchanged. *In vivo*, this was manifested as a decreased serum BA clearance in all knockout mice. In a subset of mice, NTCP deficiency resulted in markedly elevated total serum BA concentrations, mainly composed of conjugated BAs. The hypercholanemic phenotype was rapidly triggered by a diet supplemented with ursodeoxycholic acid. Biliary BA output remained intact, while fecal BA excretion was reduced in hypercholanemic *Slc10a1^−/−^* mice, explained by increased *Asbt* and *Ostα/β* expression. These mice further showed reduced *Asbt* expression in the kidney and increased renal BA excretion. Hepatic uptake of conjugated BAs was potentially affected by down-regulation of OATP1A1 and up-regulation of OATP1A4. Furthermore, sodium-dependent taurocholic acid uptake was inhibited by Myrcludex B in wild-type hepatocytes, while *Slc10a1^−/−^* hepatocytes were insensitive to Myrcludex B. Finally, positron emission tomography showed a complete abrogation of hepatic binding of labeled Myrcludex B in *Slc10a1^-/-^* mice. *Conclusion:* The *Slc10a1*-knockout mouse model supports the central role of NTCP in hepatic uptake of conjugated BAs and hepatitis B virus preS1/Myrcludex B binding *in vivo*; the NTCP-independent hepatic BA uptake machinery maintains a (slower) enterohepatic circulation of BAs, although it is occasionally insufficient to clear BAs from the circulation. (Hepatology 2015;62:207–219)

During and after a meal, bile acids (BAs) are released into the small intestine, where they facilitate the absorption of dietary fat and fat-soluble vitamins.^1^ The majority of BAs are reabsorbed in the terminal ileum and transported back to the liver through the portal circulation. At the basolateral membranes of the hepatocytes, BAs are taken up from the portal circulation, extruded across the canalicular hepatocyte membrane, and transported to the gallbladder to complete the enterohepatic circulation.^2^ Conjugated BAs are transported across the basolateral hepatic membrane through a sodium-dependent pathway, mainly mediated by the sodium-taurocholate cotransporting polypeptide (NTCP, gene name *SLC10A1*)^3^,^4^ and a sodium-independent pathway. The latter is likely mediated by members of the organic anion transporter superfamily (OATPs), which are also responsible for the hepatocellular uptake of unconjugated BAs.^5^,^6^ A liver-specific dimeric transmembrane glycoprotein,^7^ NTCP is responsible for the majority of glycine/taurine-conjugated BA uptake in primary hepatocytes.^4^ Besides its role in BA homeostasis, NTCP is able to transport sulfated steroids, thyroid hormones, and xenobiotics.^8^,^9^ Reducing hepatocellular BA uptake is considered a hepatic defense mechanism to prevent accumulation of potentially toxic BAs within hepatocytes. At the transcriptional level NTCP is regulated by cellular signaling pathways directly or indirectly involving farnesoid X receptor and small heterodimer partner.^10^ In conditions that could lead to hepatocellular BA overload, such as cholestasis, NTCP is strongly down-regulated.^11^ Recently, interest in NTCP activity and regulation and its contribution to BA homeostasis was boosted immensely when Yan and coworkers^12^ identified NTCP as the functional cellular receptor permitting hepatitis B virus (HBV) and hepatitis D virus to enter primary human liver cells. Their findings were confirmed by others,^13^ who showed that HBV binding to NTCP is mediated by the myristoylated preS1 domain of the HBV large surface (L)-protein.^14^ Virus entry mediated by NTCP is blocked by Myrcludex B, a synthetic preS1 lipopeptide derived from the HBV L-protein.^15^ The consequences of prolonged inhibition of NTCP-mediated BA transport *in vivo* are unknown. This is important to investigate since Myrcludex B inhibits NTCP-mediated BA uptake at high concentrations.^13^ Here, we describe the first genetic knockout (KO) mouse model to study the consequences of prolonged and complete absence of NTCP. Our findings underscore that NTCP plays a pivotal role in hepatic uptake of conjugated BAs *in vivo* and is a crucial receptor for the myristoylated preS1 domain of the HBV L-protein.

## Materials and Methods

More detailed information about quantification of BAs, hepatocyte culture and taurocholic acid (TCA) uptake *in vitro*, BA dynamics *in vivo*, Myrcludex imaging, and the methods described below is provided in the Supporting Information.

## Gene-Targeting Strategy and Generation of Slc10a1^−/−^ Mice

**Figure 1:**
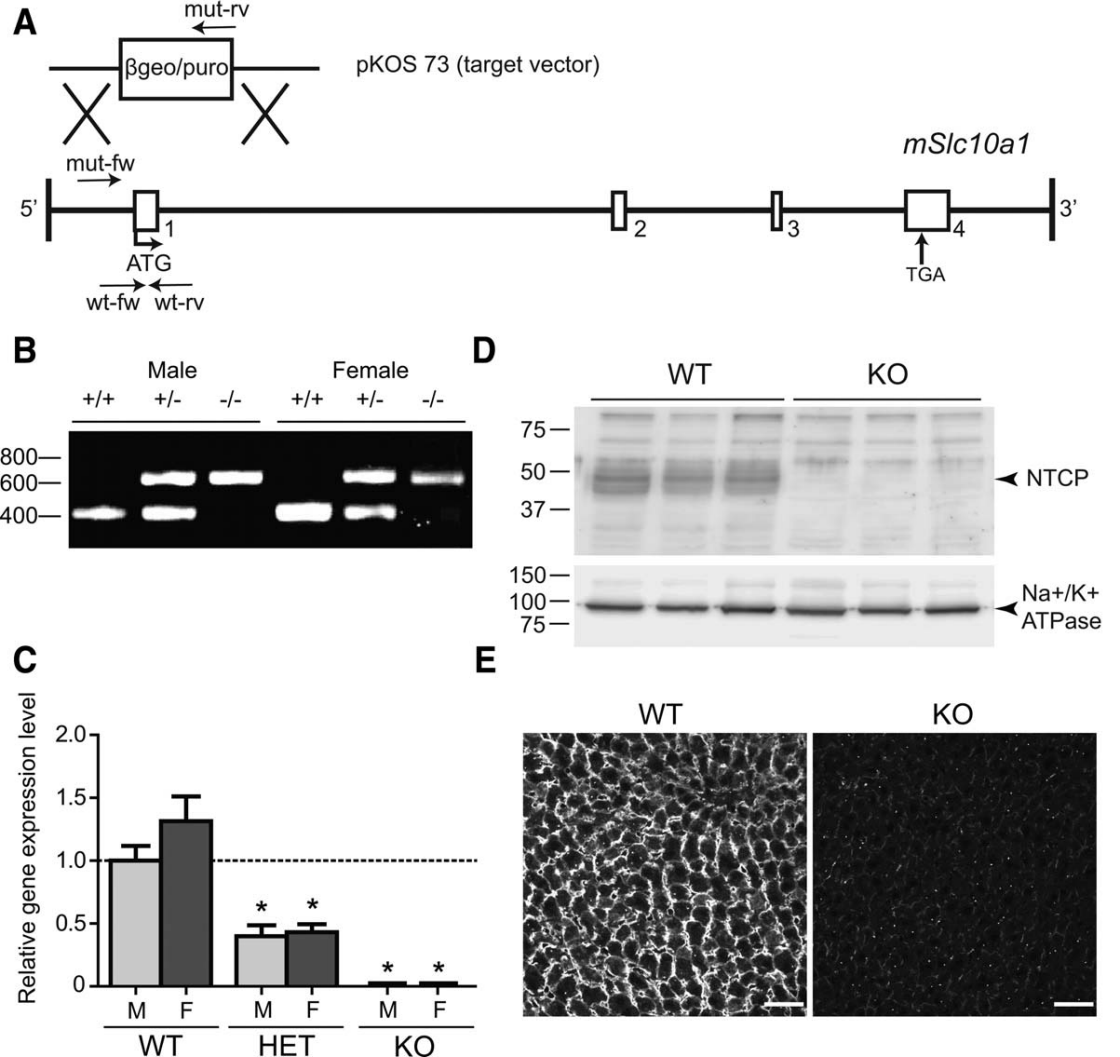
(A) Conventional knockout of *Slc10a1* in mouse embryonic stem cells by homologous recombination. A schematic representation of the mouse *Slc10a1* gene. Exon 1 was replaced by the β-galactosidase/puromycin (βgeo/puro) selection cassette located in the targeting vector (pKOS 73). Primers of the wild-type allele (wt-fw and wt-rv) and primers of the targeted allele (mut-fw and mut-rv) are shown. (B) Genomic DNA was isolated for genotyping of the offspring mice. Analysis by PCR detected fragments of the *Slc10a1* WT (412-bp) and targeted (624-bp) alleles. Homozygous WT (+/+) mice as well as heterozygous (+/−) or homozygous KO (−/−) male and female mice are shown. All PCR products were separated by 1.0% agarose gel electrophoresis. (C) *Slc10a1* mRNA expression in female and male WT, heterozygous (HET), and *Slc10a1^−/−^* (KO) mouse livers, analyzed by qRT-PCR. *Slc10a1* expression values were calculated relative to the geometric mean of *Rplp0* and *Tbp* and normalized to male WT mice (set at 1.0). Data represent mean ± SEM from six or seven male (light gray) or female mice (dark gray). *P < 0.05. (D) Levels of NTCP protein (∼45-50 kDa) in liver plasma membranes of WT and KO male mice (n – 3), analyzed by western blot. Equal volumes of protein were loaded, as confirmed by the sodium/potassium adenosine triphosphatase (∼90 kDa). (E) Immunofluorescent NTCP staining in WT and KO fresh frozen liver tissue. Scale bar – 40 μm.

Mice heterozygously lacking exon 1 of *Slc10a1* were generated by Lexicon Genetics, Inc. (The Woodlands, Texas), and obtained from Taconic (Hudson, NY). Detailed information on gene targeting (Fig. 1A) and mouse housing and feeding is provided as Supporting Information. The study design and all protocols for animal care and handling were approved by the Institutional Animal Care and Use Committee of the University of Amsterdam.

## RNA Isolation, Quantitative Real-Time Polymerase Chain Reaction, and Western Blot Analysis

Total RNA was isolated with TRIzol reagent (Invitrogen, Bleiswijk, The Netherlands). Complementary DNA was synthesized from DNAse-treated total RNA with Oligo-dT_12-18_ and Superscript II reverse transcriptase (Invitrogen). Quantitative real-time polymerase chain reaction (qRT-PCR) was carried out in a Roche Lightcycler 480 II instrument using SensiFAST SYBR No-ROX kit (Bioline, London, UK). Expression levels in each sample were normalized for the geometrical mean of two reference genes (Supporting Information). Crude mouse liver membranes were isolated by ultracentrifugation, and the pellets were resuspended in radio immunoprecipitation assay buffer (50 mM Tris-HCl [pH 8.0], 150 mM NaCl, 1% [v/v] NP-40, 0.5% [w/v] Na-deoxycholate, 0.1% [w/v] sodium dodecyl sulfate). Membrane proteins were separated on a 10% sodium dodecyl sulfate-polyacrylamide electrophoresis gel, transferred to nitrocellulose, and probed with primary antibodies as described in the Supporting Information. Equal loading was ensured by reprobing the membrane with an anti-sodium/potassium adenosine triphosphatase antibody (kind gift of J. Koenderink, Nijmegen, The Netherlands) or with an anti-β-actin antibody (Abcam; ab8226).

## Bile Secretion and TCA Elimination *In Vivo*

Bile secretion in fasted *Slc10a1^−/−^* mice was investigated by performing gallbladder cannulation and bile collection for 30 minutes after distal ligation of the common bile duct.^16^ Bile flow was determined gravimetrically assuming a density of 1 g/mL for bile. Bile output was calculated as the product of the bile flow and the bile concentration in the second 10-minute collection period. After the 30-minute depletion phase, a single bolus of 150 μmol TCA/kg mouse (including a trace amount of tritium-labeled TCA) was administered intravenously in the tail vein in 100 μL 0.9% NaCl per 20 g mouse. A heating pad maintained body temperature at 37°C. Blood samples (∼30 μL) were collected 30 minutes before TCA administration, and both blood and bile were collected at the indicated time points after TCA administration. Radioactivity in serum and bile was measured by liquid scintillation counting, and values are shown as a percentage of the total bolus (–100%).

## Peptide Synthesis, ^68^Ga Labeling, and Positron Emission Tomographic Imaging

Myrcludex B-derived myristoylated HBV preS1 peptides were synthesized on a solid-phase matrix using fluorenylmethoxycarbonyl/*t*-butyl chemistry, as described.^17^ A full description of Myrcludex B synthesis is provided in the Supporting Information. Peptides are depicted schematically in Supporting Fig. 4. For ^68^Ga labeling, 1000 μL [^68^Ga]Ga^3+^ eluate, 20 μL of a 1-mM peptide solution in dimethyl sulfoxide, 10 μL ascorbic acid solution (20% in H_2_O), and 295 μL 2.5 M sodium acetate were mixed (pH 3.6) and heated at 90°C for 15 minutes. The labeled peptide was purified using solid-phase extraction (Thermo Scientific, Schwerte, Germany), eluted (ethanol), dried, and dissolved in 60 μL dimethyl sulfoxide; and then 240 μL 0.9% NaCl was added. Mice were anesthetized with 1% sevoflurane (Abbott, Wiesbaden, Germany) and placed into an Inveon small animal positron emission tomographic (PET) scanner (Siemens, Knoxville, TN). Investigators were blinded to mouse genotypes. A dynamic microPET was performed up to 60 minutes postinjection with ^68^Ga-labeled peptides (6-27 MBq/animal in a 100-μL peptide solution in a tail vein).

## Statistical Analysis

Data are provided as the mean ± standard error of the mean. Differences between groups were analyzed using a two-tailed Student *t* test. For serum and bile kinetics, half-time (*t*_½_) and one-phase decay curves were calculated using Graphpad Prism 5. Results were considered statistically significant at *P* < 0.05.

## Results

### Generation of NTCP-Deficient Mice by Targeted KO of the *Slc10a1* Gene

Homologous recombination leading to the deletion of exon 1 in the *Slc10a1* locus (Fig. 1A) on chromosome 12, with replacement of a β-galactosidase/puromycin resistance gene cassette in embryonic stem cells and generation of heterozygous KO animals, was performed by Lexicon. Offspring that were NTCP-deficient showed normal Mendelian frequency and viability. They were fertile and showed no obvious anatomic abnormalities. Analysis by PCR identified the expected recombination by revealing the *Slc10a1* wild-type (WT, 412-bp) and targeted (624-bp) alleles in both male and female mice (Fig. 1B; primers are shown in Supporting Table 1). Analysis of liver RNA revealed that the *Slc10a1* mRNA was totally absent in the KO mice and was reduced to 39.9 ± 8.7% and 43.1 ± 6.4% in male and female heterozygous mice, respectively, compared to WT mice (Fig. 1C). This suggests a lack of transcripts or an unstable *Slc10a1* product in KO mice. Note that the primers encompass exon 4 of *Slc10a1*, which is not targeted by homologous recombination (see Supporting Table 2). Furthermore, a glycosylated form of NTCP (∼45-50 kDa; Supporting Fig. 1B) was detected in the liver homogenates of WT mice (n – 3) but was undetectable in KO mice, as shown by western blot analysis (Fig. 1D). In heterozygous mice, NTCP protein expression was approximately 90 ± 30% of the levels observed in WT mice (Supporting Fig. 1A), explaining the absence of any apparent phenotypic changes in those mice. Lack of NTCP signal in immunofluorescent staining of mouse liver confirmed the null phenotype (Fig. 1E).

### Diminished TCA Uptake and Extremely High Taurine-Conjugated Serum BA Concentrations in Adult *Slc10a1*^−/−^ Mice

**Figure 2:**
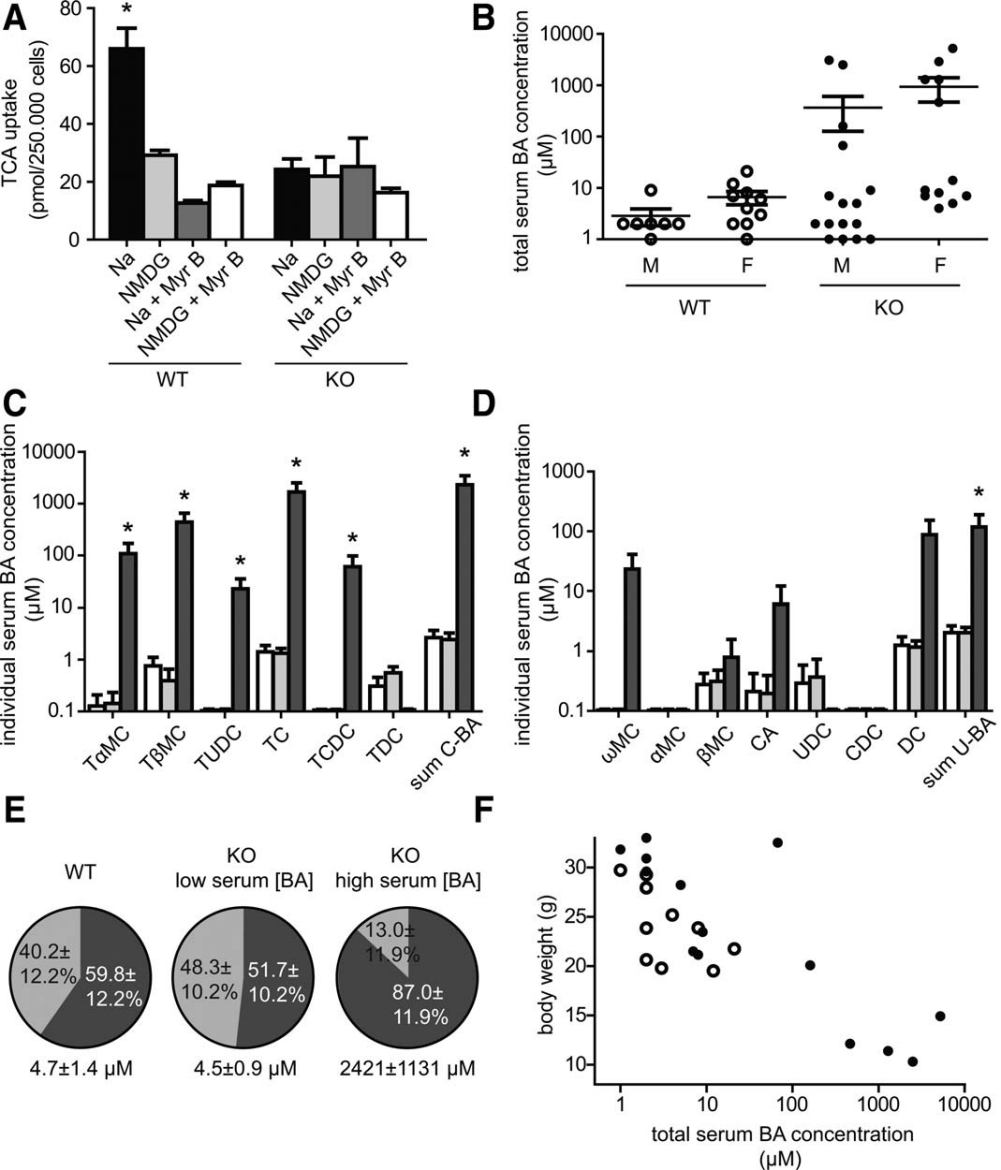
(A) Results of a TCA uptake assay in primary hepatocytes of WT and *Slc10a1^−/−^* (KO) mice. Uptake of TCA was determined after preincubation for 30 minutes in sodium-containing buffer (Na, black bar), sodium-free buffer (NMDG, light gray bar), sodium-containing buffer plus 1 μM Myrcludex B (Na + Myr B, dark gray bar), and sodium-free buffer plus 1 μM Myrcludex B (NMDG + Myr B, white bar). Bars represent the mean ± SEM for two independent experiments, each performed in triplicate. *P < 0.05. (B) Total serum BA concentrations in male and female WT and *Slc10a1^−/−^* (KO) mice. Data are given as mean ± SEM on a ^10^log scale for seven to 16 mice in each group, quantified using the total BA assay. Serum composition of (C) conjugated and (D) unconjugated BA species as quantified using high-pressure liquid chromatography. Results for WT (white bars), *Slc10a1^−/−^* mice with normal serum BA concentrations (light gray bars), and *Slc10a1^−/−^* mice with high serum BA concentrations (dark gray bars) are shown. Conjugated BA (C-BA) concentrations are the sum of tauro-α-muricholic acid, tauro-β-muricholic acid, tauroursodeoxycholic acid, TCA, taurochenodeoxycholic acid, and taurodeoxycholic acid. Unconjugated BA (U-BA) concentrations are the sum of ω-muricholic acid, α-muricholic acid, β-muricholic acid, cholic acid, UDCA, chenodeoxycholic acid, and DCA. Data are given as mean ± SEM on a ^10^log scale for three to six mice in each group. *P < 0.05 (compared to WT levels). (E) Serum BA composition of WT mice, *Slc10a1^−/−^* mice with normal serum BA concentrations, and KO mice high serum BA concentrations. Conjugated BA (dark gray) and unconjugated BA (light gray) species are shown as a percentage of total, as quantified using high-pressure liquid chromatography. (F) Total serum BA levels in relation to body weights of WT (open dots) and KO (black dots) 8-week-old mice (n – 11–14).

To assess whether *Slc10a1^−/−^* mice lose sodium-dependent BA uptake, TCA transport was assessed in sandwich cultured primary mouse hepatocytes (PMHs) from WT and *Slc10a1^−/−^* mice. Since *Slc10a1* expression is rapidly down-regulated in primary hepatocyte cultures,^18^,^19^ the TCA uptake assay was performed 3 hours after PMH seeding. Uptake increases in a linear fashion within at least 60 seconds (data not shown). Sodium-dependent TCA uptake of WT PMH was 66.0 ± 7.1 pmol/min, which decreased to 29.2 ± 1.7 pmol/min following replacement of Na^+^ with *N*-methyl-d-glucamine (Fig. 2A). This sodium-dependent TCA uptake was completely absent in *Slc10a1^−/−^* PMHs, which showed similar TCA uptake as WT mice under sodium-free conditions (24.3 ± 3.6 pmol/min). The presence of 1 μM Myrcludex B abrogated sodium-dependent TCA uptake in WT PMHs, as shown previously,^13^ but had no effect on TCA uptake under sodium-free conditions. In *Slc10a1^−/−^* PMHs, TCA uptake remained at similar levels in the sodium-dependent as well as sodium-free conditions, and this was unaffected by the addition of 1 μM Myrcludex B.

To investigate whether NTCP affects BA homeostasis, total serum BA concentrations and different BA species were measured in WT and *Slc10a1^−/−^* mice fed a standard diet. A subpopulation of NTCP-deficient mice had significantly elevated total serum BA levels, reaching millimolar concentrations (Fig. 2B). No obvious gender differences were observed for this phenotype. Total serum BA levels were in the normal range for both WT and heterozygous mice (range 1-21 μmol/L), and only WT mice were used for further comparisons. Notably, the majority of *Slc10a1^−/−^* mice (60%-75%) had normal serum BA concentrations, despite a complete lack of NTCP protein. Hypercholanemia during NTCP deficiency was defined as a total serum BA level >20 μmol/L. Both hypercholanemia and normocholanemia in *Slc10a1^−/−^* mice were preserved during the backcross to C57Bl/6J (from 50% genetically similar to 129/SvEv to >98% similar to C57Bl/6J), excluding effects of different mouse strain backgrounds. Next, the serum concentrations of individual BA species were determined by high-pressure liquid chromatography. *Slc10a1^−/−^* mice with high serum BA concentrations had significant increases in TCA, tauro-α-muricholic acid, and tauro-β-muricholic acid (Fig. 2C), which were 1673 ± 840.1 μmol/L, 107.9 ± 63.1 μmol/L, and 439.8 ± 214.3 μmol/L, respectively. Deficiency of NTCP caused a relatively mild increase in unconjugated BAs, only in mice with high serum BA concentrations, which was caused by an increase of deoxycholic acid (DCA) to 87.4 ± 65.3 μmol/L (Fig. 2D). Glycine-conjugated species were below the detection limit (0.125 μmol/L) in all mice; thus, they were not included in our results. *Slc10a1^−/−^* mice with high serum BA concentrations have a shift toward increased conjugated BA species (87 ± 12% of total versus 59.8 ± 12% in WT mice) (Fig. 2E), whereas this shift was not observed in *Slc10a1^−/−^* mice with physiological BA concentrations (51.7 ± 10% of total).

### Phenotypic Characteristics of *Slc10a1*^−/−^ Mice

Heterozygous mice carrying one intact *Slc10a1* allele were indistinguishable from WT littermates. *Slc10a1^−/−^* mice were phenotypically very similar to their littermates, except that the mice (in both the male and female groups) had reduced body weights directly postweaning (day 21) compared to littermates; and this difference was also present at 2 months of age (Table 1). The body weights were inversely correlated with total serum BA levels (Fig. 2F), and the hypercholanemic KO mice had a strongly reduced body weight. Liver morphology appeared macroscopically normal, and the ratios of liver weight to body weight were unaffected. Serum concentrations of conjugated bilirubin were moderately increased from 2.6 ± 0.6 μmol/L in WT mice to 31.4 ± 8.0 μmol/L in hypercholanemic KO mice, whereas normocholanemic KO mice did not show any elevations in conjugated bilirubin levels. Furthermore, no significant differences were found in aspartate transaminase and alanine transaminase between WT and hypercholanemic *Slc10a1^−/−^* mice (Supporting Table 3). Alkaline phosphatase increased from 22.0 ± 6.2 U/L in WT mice to 219.1 ± 38.8 U/L in hypercholanemic KO mice. These biochemical values are not indicative of extensive hepatobiliary damage as approximately 10 to 20 times higher serum levels of bilirubin and alanine transaminase are detected in severely cholestatic rodents, for instance, bile duct-ligated mice.^20^ Examination of hematoxylin and eosin-stained liver sections for all genotypes revealed no overt abnormalities. In particular, no signs of cholestasis, inflammation, or hepatocellular damage were observed in *Slc10a1^−/−^* mice (Supporting Fig. 2).

**Table 1:** Body Weight Development of WT and All *Slc10a1^-/-^* (KO) Mice*

Body Weights	WT	KO	*P* Value
Males			
2-3 weeks old	6.6 ± 0.6	7.0 ± 0.6	0.71
5-6 weeks old	21.0 ± 0.8	13.6 ± 1.6	0.001**
8-9 weeks old	24.6 ± 0.9	19.9 ± 1.4	0.02**
Females			
2-3 weeks old	7.7 ± 0.6	7.0 ± 0.6	0.41
5-6 weeks old	17.3 ± 0.4	14.7 ± 0.7	0.02**
8-9 weeks old	20.4 ± 0.4	17.8 ± 0.8	0.03**

* Male and female body weights are shown (n – 7-17 per group). Values (in grams) are given as mean ± SEM.

** *P* < 0.05.

### Normocholanemic *Slc10a1*^−/−^ Mice Fed a 0.1% Ursodeoxycholic Acid Diet Rapidly Develop Extremely High Serum BA Concentrations

**Figure 3:**
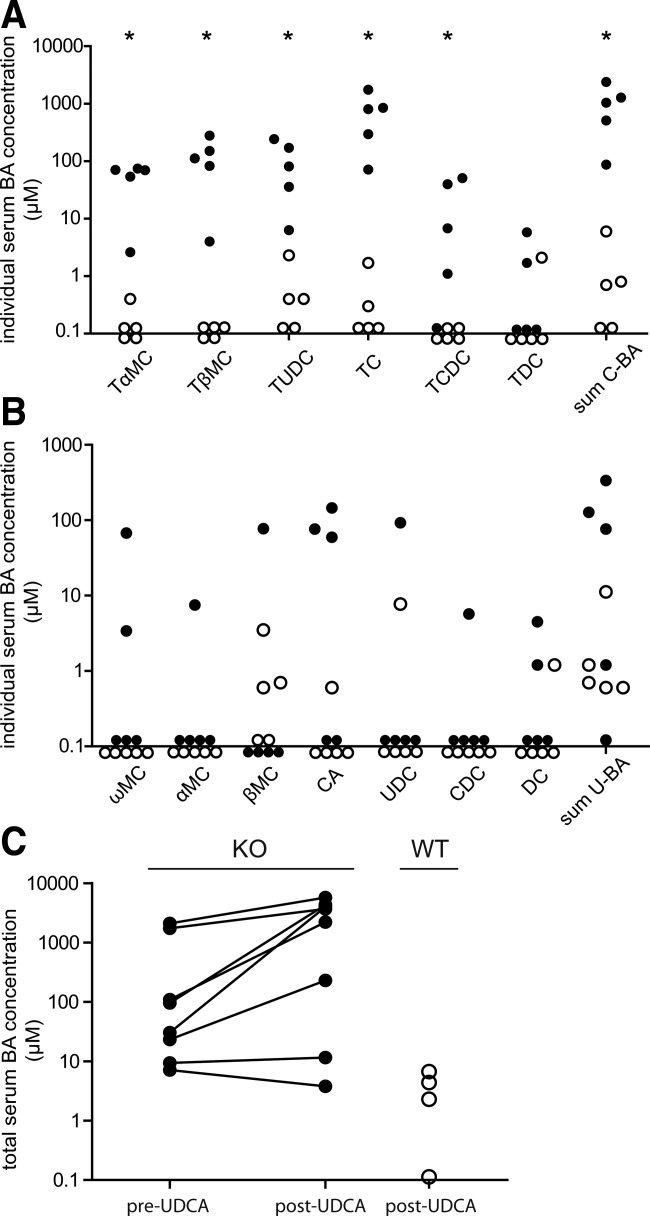
Effects of BA supplementation on serum BA concentrations. After 4 days of 0.1% UDCA supplementation, serum composition of (A) conjugated and (B) unconjugated BA species was measured in 5-week-old WT (open dots) and *Slc10a1^−/−^* (black dots) mice. Data are given as a scatter plot on a ^10^log scale from five mice in each group. *P < 0.05. (C) Total serum BA levels were measured before starting 0.1% UDCA supplementation (pre-UDCA) and after 1 week (post-UDCA) in 8- to 10-week-old *Slc10a1^−/−^* mice (black dots). As a control total serum BA levels of WT mice (open dots) were measured after 0.1% UDCA supplementation. Data are given on a ^10^log scale from four WT and eight *Slc10a1^−/−^* mice.

To challenge the hepatic BA uptake machinery during NTCP deficiency, both young and adult WT and *Slc10a1^−/−^* mice were fed for 4-8 days with 0.1% ursodeoxycholic acid (UDCA), with the rationale to increase the amount of BAs reaching the portal circulation that need to be handled by the liver. Under this condition, young *Slc10a1^−/−^* mice showed extremely high serum BA levels, which was attributed to a significant increase in conjugated BAs, mainly TCA and tauro-β-muricholic acid (Fig. 3A) but also unconjugated species tended to be elevated in a subset of mice (Fig. 3B). Experiments were repeated with adult mice, and BA levels were measured before the start of UDCA supplementation. After 1 week of 0.1% UDCA supplementation, total serum BA concentrations in adult *Slc10a1^−/−^* mice significantly increased from 514.4 ± 309.1 μmol/L to 2528 ± 792.7 μmol/L versus 3.4 ± 1.4 μmol/L in WT mice fed UDCA (Fig. 3C). Two KO mice could not be forced into hypercholanemia post-UDCA supplementation, having comparable BA levels as WT mice. Two out of eight *Slc10a1^−/−^* mice were already hypercholanemic pre-UDCA supplementation and remained extremely hypercholanemic. These findings suggest that the NTCP-independent hepatic BA uptake machinery operates close to its maximum capacity in *Slc10a1^−/−^* mice and can easily be saturated.

### Delayed Serum TCA Elimination and Biliary Excretion in *Slc10a1*^−/−^ Mice

**Figure 4:**
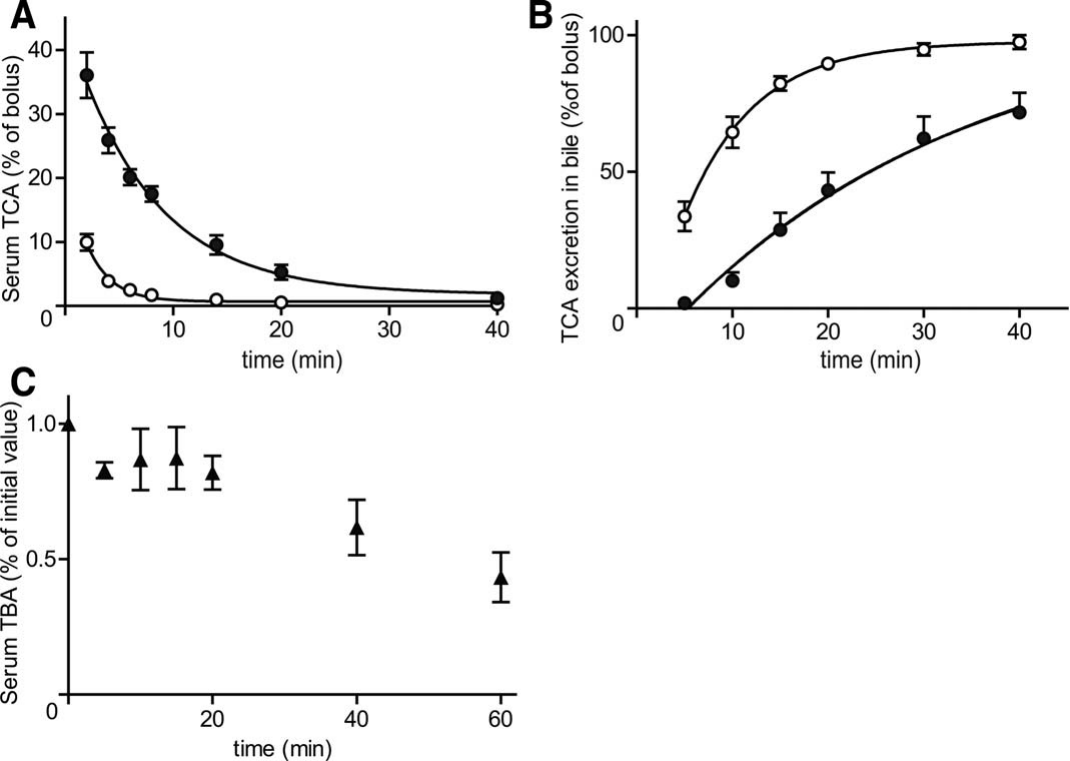
(A) *In vivo* serum TCA clearance and (B) cumulative biliary excretion in WT (open dots) and normocholanemic (black dots) *Slc10a1^−/−^* mice. The TCA kinetics after an intravenous TCA bolus (150 μmol/kg body weight) are given as a percentage of the whole bolus (–100%). Serum and bile kinetics are given as the mean ± SEM (2-40 minutes postinjection, n – 5). Mice were considered normocholanemic if the total BA concentration was <20 μM before the start of the experiment. (C) Total serum BA (TBA) clearance during hypercholanemia. Concentrations from four hypercholanemic *Slc10a1^−/−^* mice are given relative to the concentration at the start of gallbladder cannulation (set at 100%).

To examine the contribution of NTCP to (conjugated) BA kinetics *in vivo*, we investigated the clearance of conjugated BAs from serum into bile by intravenous administration of TCA in WT and both normocholanemic and hypercholanemic *Slc10a1^−/−^* mice. Serum TCA elimination *t*_½_ significantly increased from 1.5 minutes (confidence interval 1.2 ± 2.3) in WT to 5.3 minutes (confidence interval 3.9 ± 7.9) in normocholanemic *Slc10a1^−/−^* mice (Fig. 4A). Also, biliary excretion of TCA was evidently delayed in normocholanemic *Slc10a1^−/−^* mice compared to WT mice (Fig. 4B). The high dose of TCA proved to be lethal in hypercholanemic *Slc10a1^−/−^* mice shortly after injection. To assess systemic BA clearance during hypercholanemia, total serum BA concentrations were determined in time in these mice after gallbladder cannulation, without prior TCA administration. We observed a markedly delayed serum BA clearance (*t*_½_ >40 minutes; Fig. 4C).

### Biliary, Fecal, and Renal BA Excretion During NTCP Deficiency

**Figure 5:**
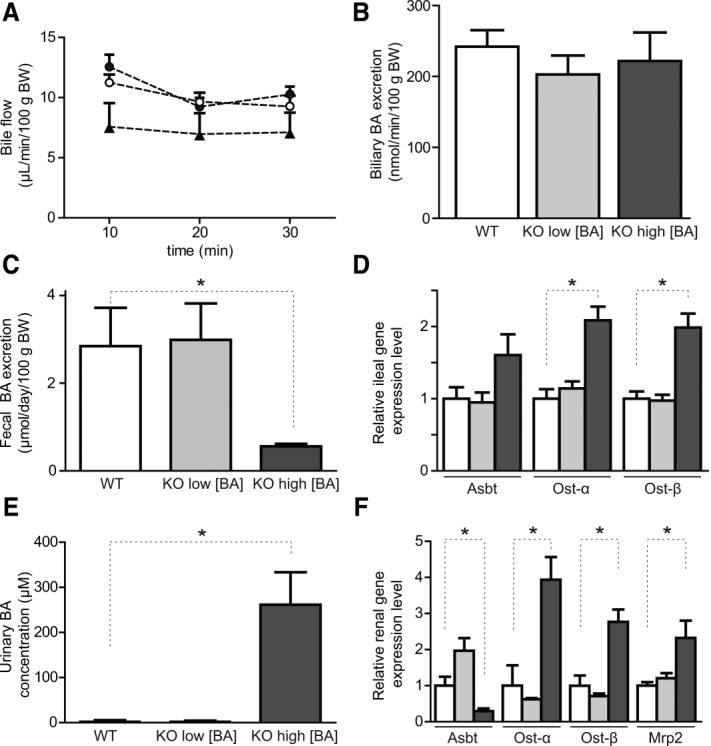
Effects of NTCP deficiency on the enterohepatic circulation. Bile was collected for 30 minutes after gallbladder cannulation and ligation of the common bile duct. (A) Bile flow (microliters per minute per 100 g body weight) is shown during this period for WT (open dots), normocholanemic *Slc10a1^−/−^* (black dots), and hypercholanemic *Slc10a1^−/−^* (black triangles) mice. (B) Bile acid output (nanomoles per minute per 100 g body weight) during the 10- to 20-minute collection period for WT (white bar), normocholanemic *Slc10a1^−/−^* (light gray bar), and hypercholanemic *Slc10a1^−/−^* (dark gray bar) mice. Data are given as mean ± SEM (n – 8-12). (C) Total fecal BA excretion (micromoles per 24 hours per kilogram of body weight) and (D) relative mRNA expression of BA transporters in the distal ileum for WT mice (white bars) and for *Slc10a1^−/−^* mice with low (light gray bars) and high (dark gray bars) serum BA concentrations, using the geometric mean of control genes *Hprt* and *Ppib*. (E) Urinary BA concentrations (μmol/L) and (F) relative mRNA expression of renal BA transporters for WT (white bars) and for *Slc10a1^−/−^* mice with low (light gray bars) and high (dark gray bars) serum BA concentrations, using the geometric mean of control genes *Rplp0* and *Hprt*. Data in (C-F) are given as mean ± SEM (n – 4-6) from 6- to 8-week-old mice. *P < 0.05 (compared to WT levels).

General BA excretion during NTCP deficiency was investigated by collecting bile during a 30-minute period. At every time point after gallbladder cannulation, no significant changes in bile flow were observed between WT and normocholanemic *Slc10a1^−/−^* mice (Fig. 5A), while hypercholanemic *Slc10a1^−/−^* mice showed a trend toward slightly decreased bile flow (*P* – 0.38). Biliary excretion of BAs was determined in the second 10-minute period after gallbladder cannulation, and no significant differences were measured between WT, normocholanemic, and hypercholanemic *Slc10a1^−/−^* mice (Fig. 5B). Hence, biliary excretion of BAs appears unaffected by NTCP deficiency.

To gain insight into BA excretion routes during NTCP deficiency, fecal and urinary BA levels were measured, together with ileal and renal mRNA expression of major BA transporters. Total fecal BA excretion significantly decreased to 0.6 ± 0.1 μmol/day/100 g body weight during hypercholanemia (Fig. 5C) compared to 2.8 ± 0.9 and 3.0 ± 0.8 μmol/day/100 g body weight in WT and normocholanemic *Slc10a1^−/−^* mice. Decreased fecal BA excretion corresponded with elevated mRNA expression of ileal *Asbt* (*P* – 0.09), *Ostα*, and *Ostβ* (Fig. 5D). Very high urinary BA concentrations were measured during hypercholanemia (Fig. 5E). Urinary BAs were mainly taurine-conjugated muricholates and cholate (data not shown). Increased renal BA excretion corresponded with decreased expression of renal *Asbt* to approximately 30% of WT and normocholanemic mice (Fig. 5F). Notably, renal expression of both the basolateral bidirectional BA transporter *Ostα/β* and the apical BA extrusion transporter *Mrp2* was significantly increased, suggesting that also tubular BA transport toward the lumen might contribute to urinary BA excretion.

### Hepatic Expression of BA-Transporting OATP Family Members

**Figure 6:**
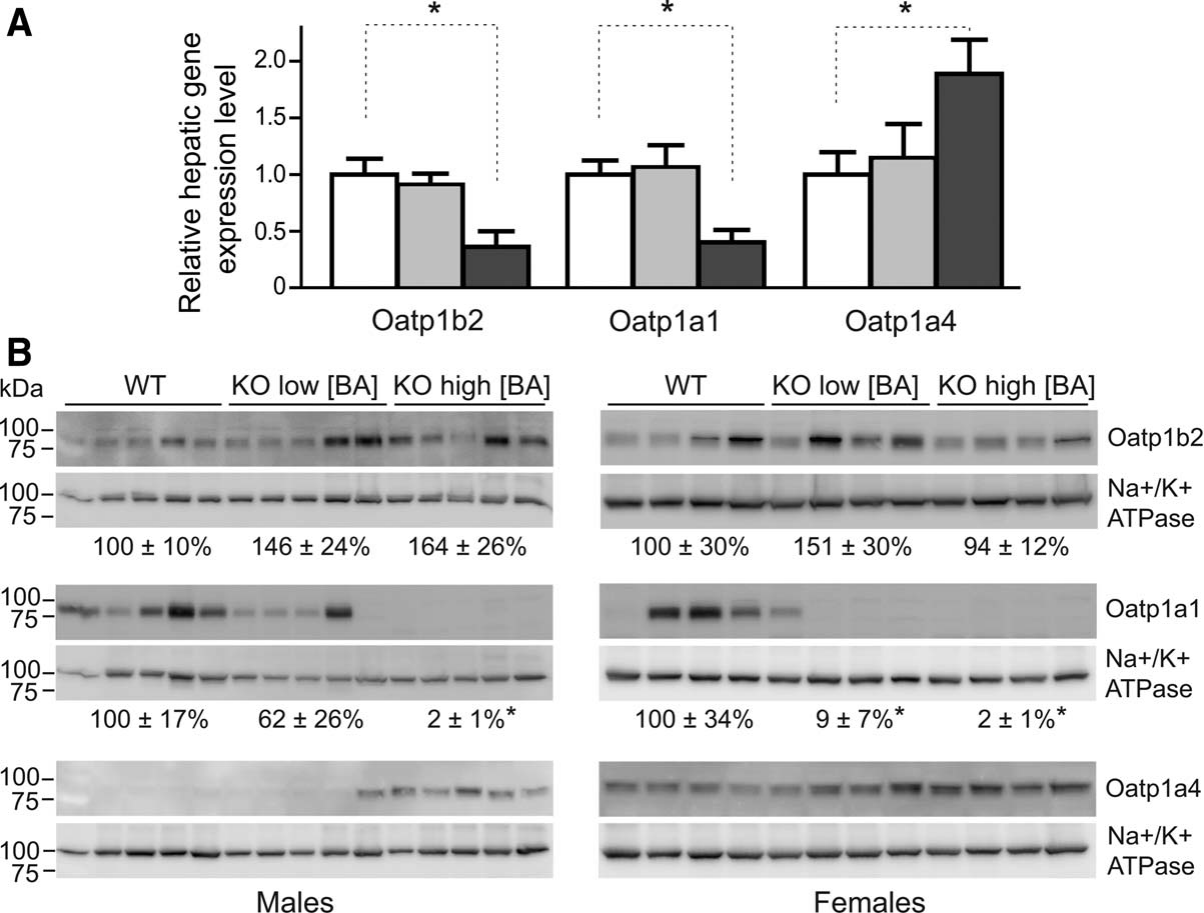
(A) *Oatp* mRNA expression as determined by qRT-PCR. Relative mRNA expression levels were calculated for WT mice (white bars) and for *Slc10a1^−/−^* mice with low (light gray bars) and high (dark gray bars) serum BA concentrations using the geometric mean of control genes *Rplp0* and *Tbp*. The two sexes were pooled equally. Values are given as mean ± SEM for four to six mice per group. (B) Western blot analysis of the OATP1B2 (∼80 kDa), OATP1A1, and OATP1A4 (both ∼85 kDa) proteins. Protein expression levels were determined relative to the sodium/potassium adenosine triphosphatase (∼90 kDa), and values are given as mean ± SEM for four or five mice per group. *P < 0.05 (compared to WT levels).

We investigated whether the basolateral hepatic BA transporters of the OATP family^21^ or the organic solute transporter α/β family compensate for the loss of NTCP, possibly explaining the normocholanemia in a subset of the *Slc10a1^−/−^* mice by restoring (conjugated) BA transport capacity. To this end, hepatic gene expression levels of mouse *Slco1a1* (*Oatp1a1*), *Slco1a4* (*Oatp1a4*), *Slco1b2* (*Oatp1b2*), and *Slc51a* (*Ostα*) were analyzed by qRT-PCR (Fig. 6A). Messenger RNA expression levels of all tested *Oatp* family members were unaffected in normocholanemic *Slc10a1^−/−^* mice, and *Ostα* mRNA was undetectable in all *Slc10a1^−/−^* mice (not shown). During hypercholanemia, *Oatp1b2* and *Oatp1a1* mRNA levels decreased to, respectively, 36 ± 14% and 40 ± 11% of WT levels; and the relative change was not caused by gender differences. On the other hand, *Oatp1a4* mRNA significantly increased to 189 ± 30%. To determine the gene expression of *Oatp* family members in response to BA supplementation, WT and hypercholanemic *Slc10a1^−/−^* mice were analyzed post-UDCA supplementation. The *Oatp* family members were regulated in the same pattern as during spontaneous hypercholanemia (Supporting Fig. 3). Consistent with the mRNA expression pattern, the OATP1A1 protein was virtually lost during hypercholanemia, while the OATP1A4 protein was significantly up-regulated (in both genders; Fig. 6B). Notably, OATP1A1 (male predominant) and OATP1A4 (female predominant) had gender-regulated protein expression levels. In contrast, *Oatp1b2* mRNA and protein expression did not correlate as OATP1B2 protein expression remained stable during NTCP deficiency. These results were opposite to the gene expression levels, suggesting that posttranscriptional regulation occurs, which has been described for Oatp isoforms by Rippin et al.^19^

### Abrogation of Myrcludex B Accumulation in the Livers of *Slc10a1*^−/−^ Mice

**Figure 7:**
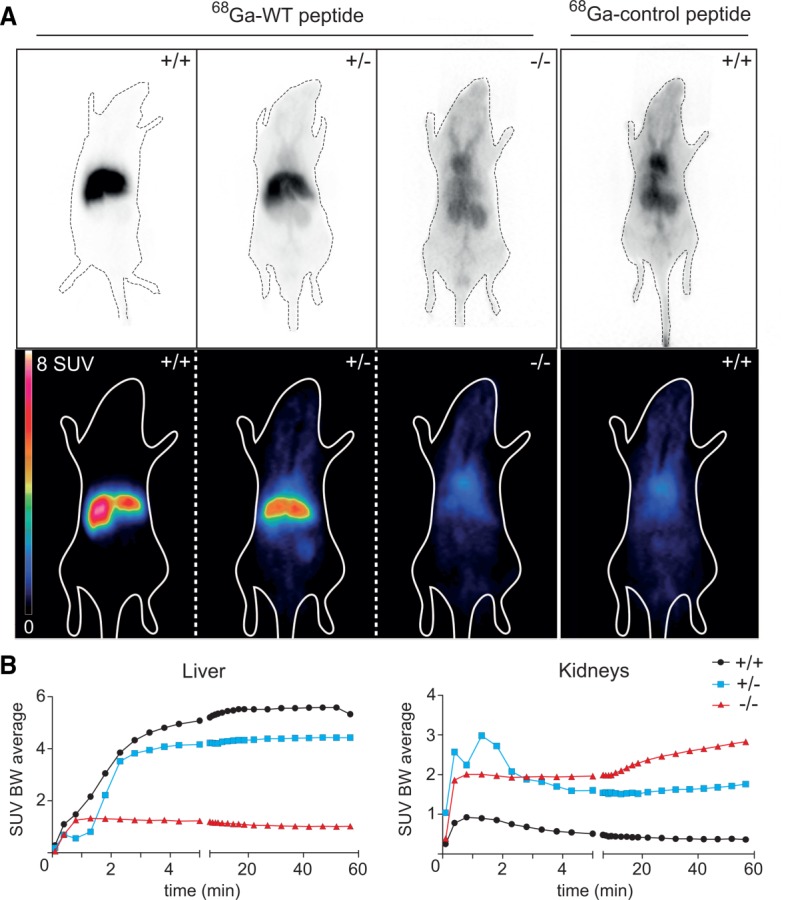
Myrcludex B-derived peptide binding assessed by microPET. (A) Female WT (+/+, first panel), heterozygous (+/-, second panel), and homozygous *Slc10a1^−/−^* (-/-, third panel) mice were imaged postinjection of ^68^Ga-WT peptide or ^68^Ga-control peptide (fourth panel). (Top) Uncorrected planar projection, (bottom) coronal PET section. (B) Time activity curves of ^68^Ga-WT peptide in liver and kidney of WT (black line with dots), heterozygous (blue line with squares), and *Slc10a1^−/−^* (red line with triangles) male mice during 60 minutes postinjection. Abbreviation: SUV BW, standardized uptake value body weight.

We evaluated the distribution of radiolabeled Myrcludex B-derived lipopeptide after intravenous injection in WT, heterozygous, and *Slc10a1^−/−^* mice. To this end, we developed peptides suitable for PET imaging containing either the Myrcludex B sequence (^68^Ga-WT peptide) or a mutation in the residues required for binding (^68^Ga-control peptide). The functionality of the peptides for liver targeting was confirmed in WT mice (Supporting Fig. 4)). As previously shown,^22^ Myrcludex B-derived peptides specifically enrich in the liver of WT mice, while mutations in the essential receptor binding residues of the peptide lead to a loss of hepatocyte binding. Crucially, livers of *Slc10a1^−/−^* mice had virtually no accumulation of radiolabeled Myrcludex B signal and livers of heterozygous mice had reduced accumulation (60%-70% compared to WT) (Fig. 7A). The distribution pattern of the ^68^Ga-WT peptide in *Slc10a1^−/−^* mice was identical to the pattern observed in WT mice injected with the ^68^Ga-control peptide. Within minutes, the WT peptide enriched in the liver of WT mice, whereas in *Slc10a1^−/−^* mice the detected signal within the liver remained at a constantly low level, which represents the blood flow through the organ (Fig. 7B, left panel). Non-liver-bound peptide in *Slc10a1^−/−^* mice accumulated in the kidneys (Fig. 7B, right panel), where it was eliminated from the circulation. No gender differences were observed. Thus, *Slc10a1^−/−^* mice lost the ability to bind Myrcludex B in the liver.

## Discussion

The present study shows the first characterization of *Slc10a1*-KO mice and illustrates the importance of this liver-specific transporter for the hepatic uptake of conjugated BAs and as a receptor for Myrcludex B, a synthetic preS1 lipopeptide derived from the HBV L-protein.

We describe the consequences of complete and prolonged absence of NTCP functionality *in vivo*. Recently, a 5-year-old patient was described with homozygosity for NTCP p.Arg252His, a mutation that strongly affects NTCP targeting to the plasma membrane.^23^ This girl showed persistent hypercholanemia without any sign of liver injury or pruritus. Other NTCP genetic variants were previously described that result in impaired BA transport capacity (e.g., p.Ser267Phe^24^), but no phenotype is yet described to associate with these variants. Our data on *Slc10a1^−/−^* mice support these findings as 30%-35% of the adult mice had markedly elevated total BA levels in serum and strongly reduced body weight. In line with the function of NTCP as a sodium-dependent transporter of mainly conjugated BAs, these mice mainly showed serum accumulation of taurine-conjugated BAs. Despite the reduced body weights and a strongly reduced capacity to remove conjugated BAs from the circulation in *Slc10a1^−/−^* mice, no overt liver damage was observed. Moderate elevations in serum alkaline phosphatase and bilirubin levels were only present in hypercholanemic *Slc10a1^−/−^* mice, and many *Slc10a1^−/−^* mice were normocholanemic. This relatively mild phenotype is in line with that of the single NTCP-deficient patient described to date. The distribution of the mice across these two groups (normocholanemic versus hypercholanemic) persisted over all generations, while the mice were backcrossed to a C57Bl/6J genetic background. We also observed temporal normalization of the serum BA concentration as well as mature onset of hypercholanemia, further excluding mouse genotype/strain effects.

We hypothesize that the sodium-independent hepatic uptake machinery is able to sustain the enterohepatic cycle of (conjugated) BAs, but in a subset of *Slc10a1^−/−^* mice the flux of BAs can (temporarily) exceed the maximum capacity of the liver to effectively clear the portal circulation from BAs. In line with this hypothesis is our finding that *Slc10a1^−/−^* mice accumulate high amounts of BAs in the circulation if they are challenged with 0.1% UDCA, and this hypercholanemia is inducible in *Slc10a1^−/−^* mice with normal or minimally elevated serum BA concentrations. Notably, this increase of serum BAs included multiple (mostly conjugated) BA species and not only (T)UDCA, which is in line with a saturated uptake machinery. Furthermore, these findings suggest that the enterohepatic circulation is still sustained as UDCA is conjugated and slightly increased levels of unconjugated BAs were measured, including cholic acid and DCA. As DCA itself seems to be a poor substrate for NTCP,^25^,^26^ increased serum DCA could point to a competitive inhibition of the OATP family members by conjugated BAs, combined with the loss of OATP1A1 protein. A mild conjugated hyperbilirubinemia was observed in hypercholanemic *Slc10a1^−/−^* mice, without evident cholestatic damage. This observation could support the hypothesis of competitive inhibition of OATP isoforms as the total *Oatp1a/1b*-KO mouse showed a 40-fold increase of serum conjugated bilirubin.^27^

Hypercholanemic *Slc10a1^−/−^* mice display interesting changes in expression of hepatic, ileal, and renal BA transporters. Also notable is that no changes in *Oatp* mRNA levels are detected in normocholanemic *Slc10a1^−/−^* mice, suggesting that compensatory up-regulation of these transporters is not occurring in NTCP-deficient mice. In line with this finding, *in vitro* TCA uptake of PMHs under sodium-free conditions is similar between WT and normocholanemic *Slc10a1^−/−^* mice. In contrast, *Oatp1a1* mRNA and protein levels decreased in hypercholanemic *Slc10a1^−/−^* mice, whereas *Oatp1a4* mRNA and protein levels slightly increased. Although it is presently unclear if the changes in OATP expression are due to or a cause of the hypercholanemic phenotype, absence of OATP1A1 could further reduce the hepatic uptake capacity for conjugated BAs,^5^ contributing to the hypercholanemia. In contrast, up-regulation of OATP1A4 could contribute to systemic BA clearance during NTCP deficiency. Fecal BA excretion certainly does not provide a likely compensatory mechanism to reduce hypercholanemia during NTCP deficiency, considering the enhanced *Asbt* expression and strongly reduced fecal BA excretion. On the other hand, enhanced renal BA excretion is the most likely route of BA elimination when hepatic BA uptake capacity reaches its maximum, thereby attenuating the circulatory BA overload. This could be mechanistically confirmed by a reduced renal *Asbt* expression. Interestingly, renal mRNA expression of *Ostα/β* and *Mrp2* was increased, possibly allowing excretion of BAs into the ductular lumen as organic solute transporter α/β has bidirectional transport properties. This hypothesis as well as the underlying mechanisms for sensing changes in BA dynamics and modulating transporter expression will require further investigation.

Recently, NTCP was identified as the receptor for HBV/hepatitis D virus entry, using a proteomic approach to identify proteins that bind the preS1 domain of the HBV L-protein.^12^ This finding could explain many of the characteristics of HBV, including its specificity to infect hepatocytes (hepatotropism), as these are the only cells that express NTCP. The rapid loss of infectability of primary hepatocytes and the total lack of virus binding in virtually all cell lines could now be linked to down-regulation of NTCP upon isolation of primary cells and absence of NTCP expression in all cell lines except differentiated HepaRG cells. The myristoylated preS1 subdomain of the HBV L-protein specifically binds to the liver in many (also non-HBV-susceptible) animals, including mice.^22^ This exclusive liver targeting makes Myrcludex B a very interesting potential drug with high hepatocyte specificity. Using the *Slc10a1-*KO model, we here show that NTCP is essential for Myrcludex B binding *in vivo*, with a total lack of liver association in *Slc10a1^−/−^* mice, indicating that association with NTCP mediates the liver specificity of Myrcludex B and supporting a role for NTCP in HBV hepatotropism.

Taken together, this report is the first to describe and characterize a murine *Slc10a1*-KO model in order to better understand the physiological relevance of this BA transporter. The data presented in this study show the importance of NTCP for sodium-dependent hepatic BA uptake *in vivo* and for hepatic binding of Myrcludex B, a peptidic inhibitor of HBV entry.

## Abbreviations

BA: bile acid
DCA: deoxycholic acid
HBV: hepatitis B virus
KO: knockout
L-protein: large surface protein
NTCP: sodium taurocholate cotransporting polypeptide
OATP: organic anion transporting polypeptide
PET: positron emission tomography
PMH: primary mouse hepatocyte
qRT-PCR: quantitative real-time polymerase chain reaction
t_½_: *half-time*
TCA: taurocholic acid
UDCA: ursodeoxycholic acid
WT: wild type
