# Supplementary material for: Impaired uptake of conjugated bile acids and hepatitis b virus pres1-binding in na+-taurocholate cotransporting polypeptide knockout mice
Source: Hepatology. 2015 May 8;62(1):207–19. doi: 10.1002/hep.27694 (PMC4657468; doi:10.1002/hep.27694)
Supplement: Supplementary file 1 [file hep0062-0207-sd1.pdf]

Supplementary Figure 1

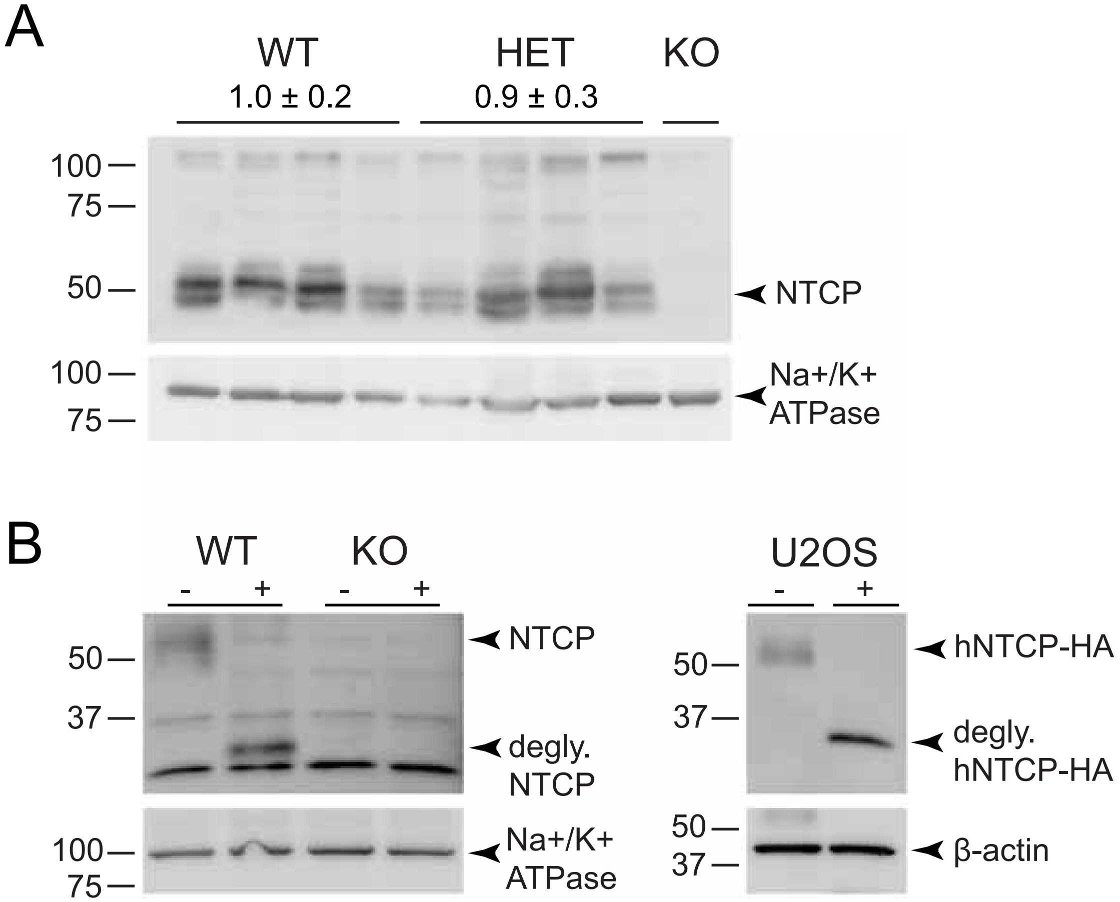

Supplementary Figure 2

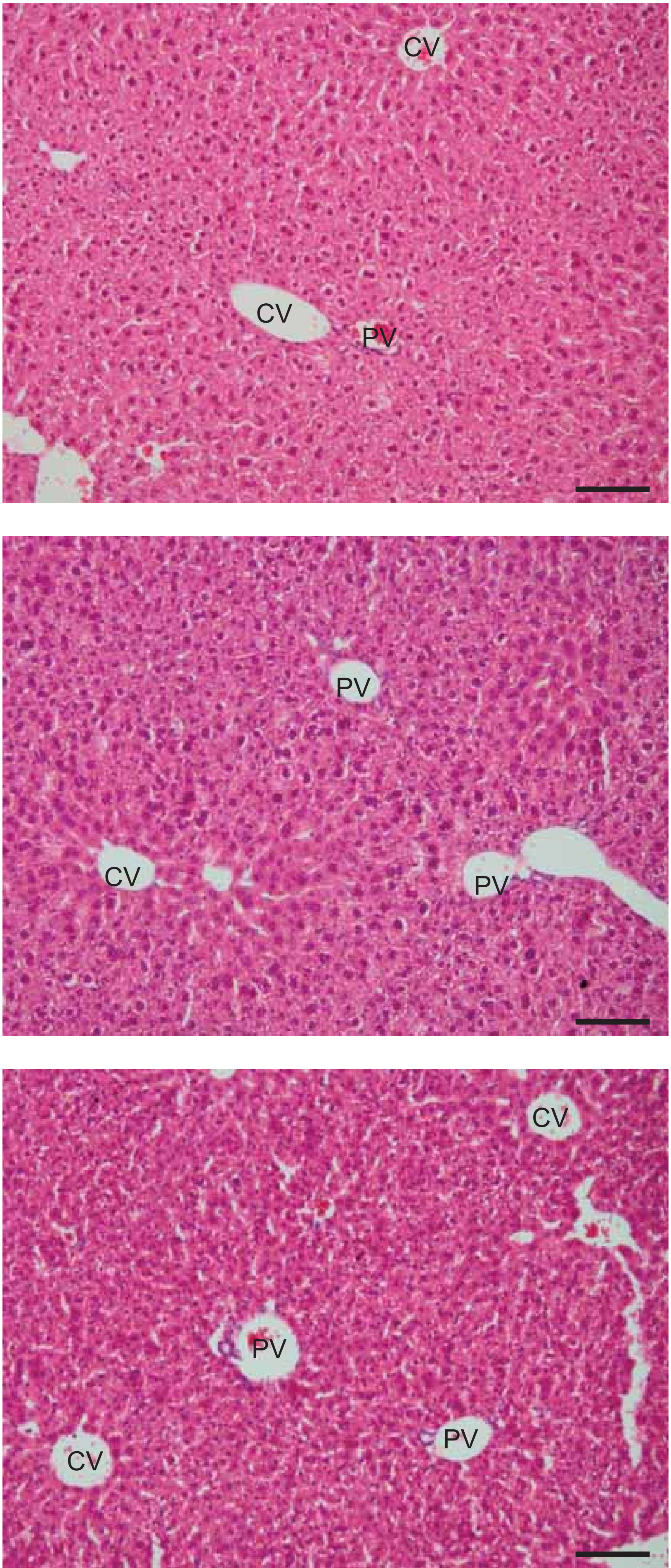

WT

KO low serum [BA]

KO high serum [BA]

Supplementary Figure 3

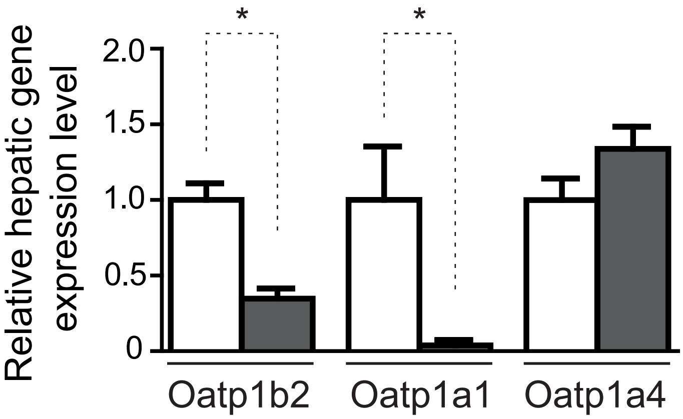

Supplementary Figure 4

A

^{68}Ga-WT peptide (myr-K(^{68}Ga-DOTA)-HBVpreS3-48y)

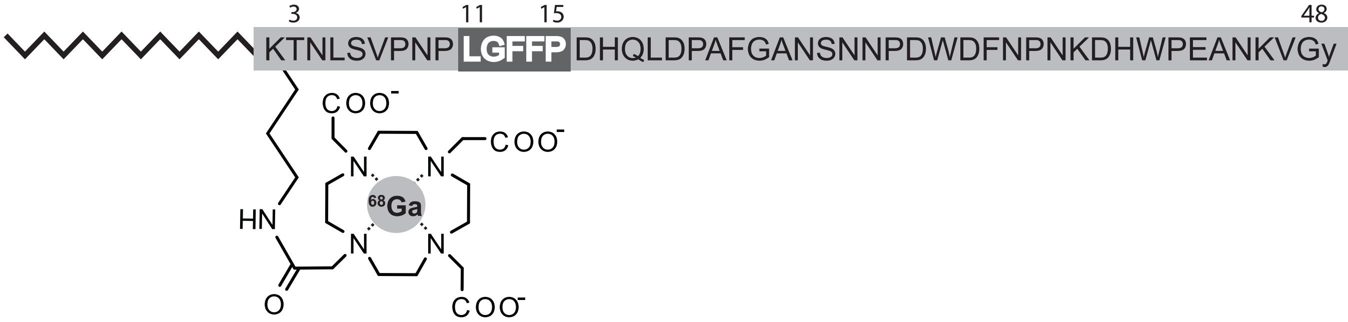

^{68}Ga-control peptide (myr-K(^{68}Ga-DOTA)-HBVpreS3-48y Ala11-15)

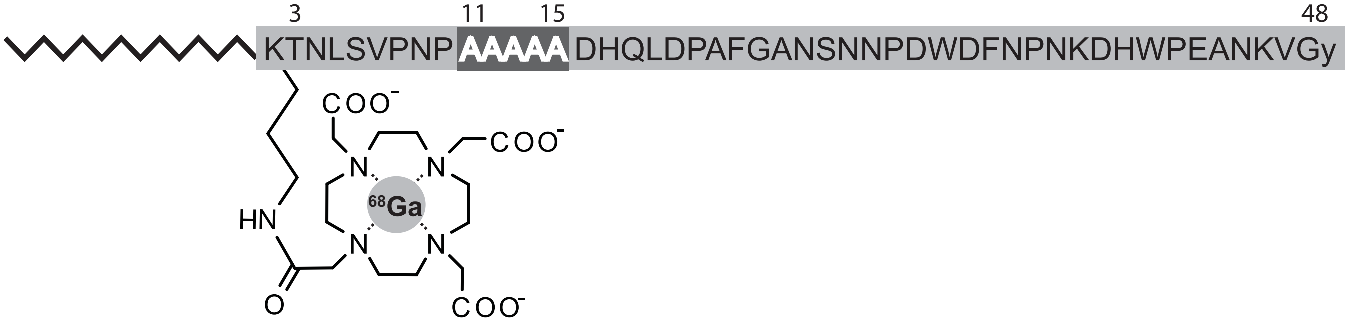

B

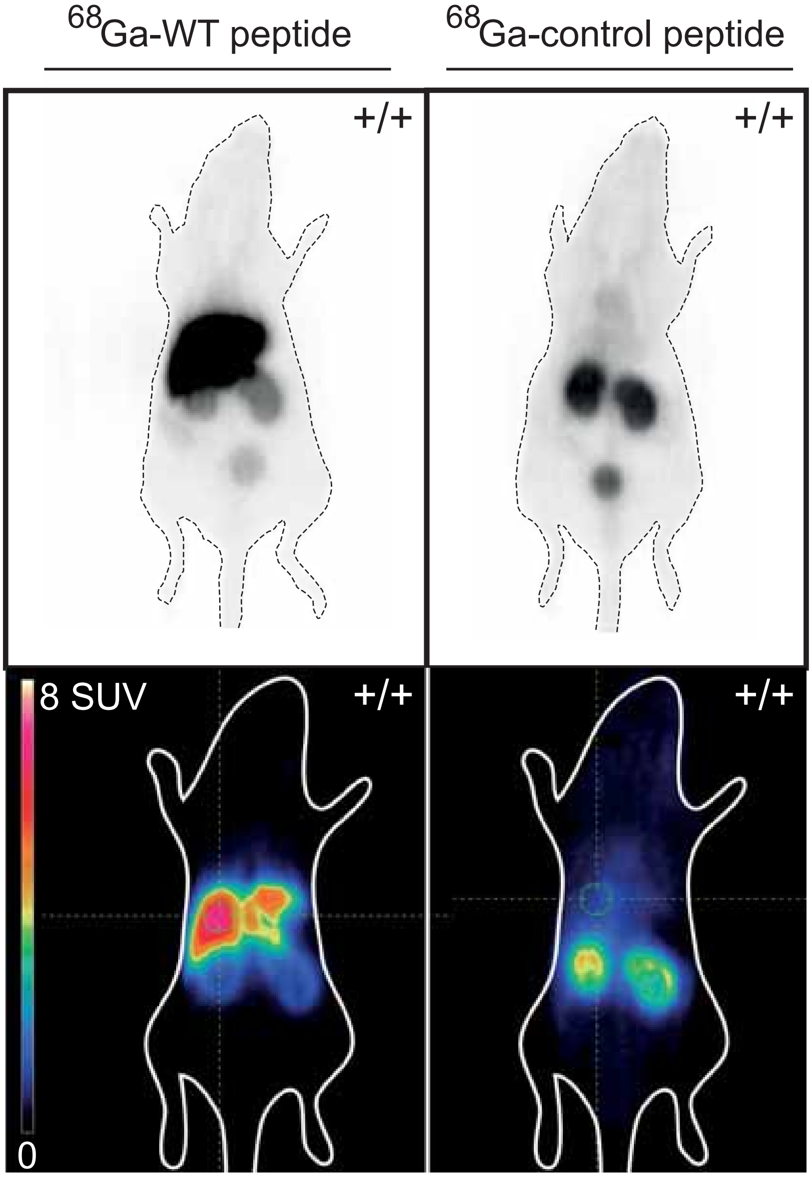

Supplementary materials & methods

Gene-targeting strategy and generation of Slc10a1$^{-/-}$ mice

Mice heterozygously lacking exon 1 of *Slc10a1* were generated by Lexicon Genetics Incorporated (The Woodlands, Texas, US), and obtained via Taconic (Hudson, NY, US). Exon 1 of *Slc10a1* was replaced using a targeting vector consisting of 5' (7.7-kb) and 3' (1.3-kb) sequence homology arms flanking a β-galactosidase/puromycin (βgeo/puro) selection cassette, as shown in figure 1A. Experiments were performed on 6 to 10 week-old *Slc10a1*$^{-/-}$ male and female mice using littermate controls, unless stated otherwise. All mice were kept on a 12h light-dark cycle and received a standard chow (SDS, Witham, England) and water *ad libitum*. In one experiment, 0.1% (w/w) ursodeoxycholic acid (UDCA; Sigma-Aldrich) was supplemented to standard chow for 4-8 days. The study design and all protocols for animal care and handling were approved by the Institutional Animal Care and Use Committee of the University of Amsterdam.

RNA isolation and qRT-PCR

Total RNA was isolated from approximately 50 mg of liver, terminal ileum and kidney with TRIzol reagent (Invitrogen, Bleiswijk, The Netherlands). RNA integrity was assessed by agarose gel electrophoresis and spectrophotometrically at 260 nm using a Nanodrop 1000 (Thermo Scientific, Wilmington, US). Five-hundred nanograms of total RNA were treated with DNAse (Promega, Madison, US) and first-strand cDNA was synthesized with Oligo-$dT_{12\text{-}18}$ and Superscript II reverse transcriptase (Invitrogen). Quantitative real-time PCR (qRT-PCR) was carried out in a Roche Lightcycler 480 II instrument using SensiFAST SYBR No-ROX kit (Bioline, UK) and was analysed using LinRegPCR 12.5 software. Expression levels in each sample were normalized for the geometrical mean of two reference genes (supplementary table 2 shows qPCR primer sequences).

Western Blot analysis

Crude mouse liver membranes were isolated by pestle homogenization on ice in hypotonic lysis buffer (10 mM KCl, 1.5 mM $MgCl_2$, 10 mM Tris-HCl pH 7.4) supplemented with protease inhibitor cocktail (Complete, Roche, Mannheim, Germany). Liver homogenates were subjected to ultracentrifugation (200,000 x g, 45 minutes) and the pellets were resuspended in RIPA buffer (50 mM Tris-HCl, pH 8.0, 150 mM NaCl, 1% (v/v) NP-40, 0.5% (w/v) Na-deoxycholate, 0.1% (w/v) SDS). Samples were treated with PNGase F according to the manufacturer's protocol (New England Biolabs), with the exception that denaturation of the samples was performed at 37 °C for 5 min. Membrane proteins (50 µg/lane) were separated on a 10% SDS-polyacrylamide electrophoresis gel, transferred to nitrocellulose and probed with rabbit anti-mouse NTCP or anti-rat OATP1A1, anti-mouse OATP1A4 (Santa Cruz: sc-18436), or anti-mouse OATP1B2 (kind gift of R. B. Kim, London, Canada) antibodies. As a positive control for NTCP, U2OS cells (human bone osteosarcoma epithelial cells) stably transfected with hNTCP-HA were analysed by Western blot, as previously described (1). Equal loading was ensured by reprobing the membrane with an anti-sodium/potassium ATPase antibody (kind gift of J. Koenderink, Nijmegen, the Netherlands) or with an anti-β-actin antibody (Abcam; ab8226). Immune complexes were detected with a horseradish peroxidase-conjugated secondary antibody (Biorad), visualized using enhanced chemiluminescence detection reagent (Lumi-light, Roche) and detected using ImageQuant LAS 4000 (GE Healthcare). Densitometry was performed in ImageJ software.

Histology and immunohistochemistry

Livers were fixed in 4% PFA overnight, dehydrated in 70% ethanol, and paraffin-embedded. For routine histological tissue examination, hematoxylin-eosin stainings were investigated in wild-type and *Slc10a1*$^{-/-}$ mice. For specific NTCP staining, small cubes of fresh frozen liver tissue were embedded in OCT (Agar Scientific), and 7 µm cryosections were fixed in 100% acetone for 10 minutes. Slides were blocked with 5% (v/v) normal goat serum in PBS + 0.05% (v/v) tween-20 (blocking buffer) for 1 hour, followed by incubation with rabbit anti-

mouse NTCP antibody in blocking buffer for 1 hour. After washings, the slides were incubated with goat anti-rabbit Alexa 568 IgG. Images were captured with a Leica SP8X confocal microscope.

Primary hepatocyte isolation and sandwich culture

Primary mouse hepatocytes (PMHs) were isolated from wild-type and *Slc10a1*$^{-/-}$ mice by a 2-step collagenase perfusion method. The liver was perfused through the portal vein with Ca^{2+}-free Krebs/Henseleit-bicarbonate buffer (pH 7.4) saturated with O_2/CO_2 (95:5 v/v) at 37 °C for 10 min, followed by perfusion for an additional 10 min with 20 mg collagenase type IV (0.5 mg/mL) and $CaCl_2$ (1.7 µmol/L). The softened liver was transferred to a sterile plastic dish, and cells were dispersed with a coarse comb in Ca^{2+}-containing Krebs/Henseleit-bicarbonate buffer (pH 7.4), and filtered through nylon gauze. The filtrate was washed and centrifuged three times 3 min at 60 x g, with a final wash in buffer containing 1% (w/v) BSA. Routinely, 30-40 million cells were obtained with viability of at least 85%, as determined by trypan blue exclusion. Next, cells were plated in Dulbecco's modified Eagle's medium (DMEM) supplemented with 2 mM L-glutamine, 100 U/L penicillin/streptomycin and 5% FCS at 250,000 cells per well in 24-wells plates pre-coated with rat-tail collagen type I (1,5 mg/ml, pH 7.4, BD Biosciences). After 3 hours for attachment at 37 °C, a second collagen layer was added to obtain a sandwich configuration, as described by Annaert (2), and cells were cultured another hour in DMEM supplemented with 2 mM L-glutamine and 100 U/L penicillin/streptomycin.

Taurocholic acid uptake assay

After a 30-minute pre-incubation at 37 °C in sodium-containing uptake buffer (136 mM NaCl, 5 mM KCl, 1.1 mM KH_2PO_4, 1 mM $MgCl_2$, 1.8 mM $CaCl_2$, 10 mM D-Glucose, 10 mM HEPES, pH 7.4) with and without Myrcludex B (1 µM), PMHs were incubated at 37 °C for 1 minute with uptake buffer containing 10 µM TCA of which a trace amount (0.1 µM) was tritium-labelled ([3H]TCA, Perkin Elmer, Groningen, The Netherlands). To assess sodium-

dependence of the TCA uptake, NaCl was replaced with equimolar N-methyl-D-glucamine (NMDG). Cells were washed four times with ice-cold PBS buffer and lysed with milliQ water containing 0.05% (w/v) SDS. Radioactivity in the lysates was measured by liquid scintillation counting. Data are presented of two independent PMH isolations, each condition performed in triplicate. All values for TCA uptake were corrected for nonspecific binding by subtracting signal determined in control dishes without cells.

Quantification of bile acids

Feces were collected (24 h) from individually housed mice (to avoid coprophagy), lyophilized and weighed. The dried stools were homogenized and BAs were extracted in 50% (v/v) t-butanol (Merck). Total BA concentrations in feces, urine and serum were quantified with the Total Bile Acid Assay kit (Diazyme Laboratories, Poway, US) by measuring absorbance at 410 nm using a NOVOstar analyzer (BMG-Labtech, Offenburg, Germany). Concentrations of different BA species in serum were determined by reverse-phase HPLC. For HPLC, serum was deproteinized by adding dropwise 5 volumes of acetonitrile (Biosolve, Valkenswaard, The Netherlands), followed by vortex-mixing, centrifugation at 12,000 x g for 10 min and vacuum-drying of the supernatant. Serum BAs were solubilized in 25% methanol. 100 µl was applied to a Hypersil C18 (3 µm, 15 cm HPLC column, Thermo Scientific, Breda, The Netherlands). Starting eluent consisted of 6.8 mM ammonium formate (pH 3.9), followed by several steps of linear gradients to different concentrations of acetonitrile (Biosolve, Valkenswaard, The Netherlands). Detection was performed using a Nano Quantity Analyte Detector QT-500 (Quant technologies, Blaine, US). Quantification of the different BA species was performed using calibration curves for all BA species.

Bile secretion and TCA elimination in vivo

To investigate bile secretion in fasted *Slc10a1*$^{-/-}$ mice, gall bladder cannulation and bile collection was performed. Bile was collected in aliquots every 10 minutes for 30 minutes after distal ligation of the common bile duct, as described in (3). Bile flow was determined

gravimetrically assuming a density of 1 g/mL for bile. Bile output was the calculated as the product of the bile flow and the bile concentration in the second 10-minute collection period. After the 30 minutes depletion-phase, a single bolus of 150 µmol TCA / kg mouse (including a trace amount of tritium-labelled TCA) was administered intravenously in the tail vein in 100 µL 0.9% NaCl per 20 g mouse. A heating pad maintained body temperature at 37 °C. Blood samples (~30 µL) were collected at 30 minutes before TCA administration and both blood and bile were collected at the indicated time points after TCA administration. Radioactivity in serum and bile was measured by liquid scintillation counting, and values are shown as a percentage of the total bolus (=100%). During the gall bladder cannulation experiment without prior TCA administration, blood was collected directly before bile collection.

Peptide synthesis, ^{68}Ga-labelling and PET-imaging

Myrcludex B-derived myristoylated HBV preS1 peptides were synthesized on a solid phase matrix using fluorenylmethoxycarbonyl/*t*-butyl (Fmoc/*t*Bu) chemistry, as previously described (4). For selective coupling of DOTA to the N-terminal lysine side chain on the solid phase, the building block Fmoc-Lys(Aloc)-OH was used. After myristoylation the Aloc orthogonal protecting group was selectively removed and the peptide was reacted with DOTA-DFP, as previously described (5). Following cleavage from the resin, the peptides were purified using preparative high-pressure liquid chromatography (HPLC) and analysed by mass spectrometry (WT-peptide myr-K(DOTA)HBVpreS3-48y; $m/z_{calculated}$: 6015.9283; $m/z_{observed}$: 6016.0201; control-peptide myr-K(DOTA)HBVpreS3-48y Ala11-15; $m/z_{calculated}$: 5809.8188; $m/z_{observed}$: 5809.9182). Peptides are depicted schematically in supplementary figure 4. For ^{68}Ga-labelling, 1000 µl [^{68}Ga]Ga^{3+} eluate, 20 µl of a 1 mM peptide solution in DMSO, 10 µl ascorbic acid solution (20% in H_2O) and 295 µl 2.5 M sodium acetate were mixed (pH 3.6) and heated at 90 °C for 15 minutes. The labelled product was purified using solid phase extraction (Thermo Scientific, Schwerte, Germany). The peptide was eluted from the cartridge with 2 ml ethanol. The ethanol was evaporated, the peptide dissolved in 60 µl DMSO and 240 µl of 0.9% NaCl were added. The solution was filtered with a 0.22 µM sterile

filter prior to injection. Mice were anesthetized with 1% sevoflurane (Abbott, Wiesbaden, Germany) and placed into an Inveon small animal PET scanner (Siemens, Knoxville, US) in a prone position. Investigators were blinded to mouse genotypes. A dynamic microPET was performed up to 60 min post-injection with ^{68}Ga-labelled peptides (6 to 27 MBq/animal in a 100 µl peptide solution in a tail vein). For pretesting of the peptides, female NMRI mice 6 to 8 weeks of age were injected with ^{68}Ga-labelled peptides and static images were taken from 60-80 min post-injection.

Statistical analysis

Data are provided as the mean ± standard error of the mean. Differences between groups were analysed using 2-tailed Student's t-test. For serum and bile kinetics, half-time ($t_{1/2}$) and one-phase decay curves were calculated using Graphpad Prism 5. Statistical significance was considered at $p < 0.05$.

References

1. Bijsmans IT, Bouwmeester RA, Geyer J, Faber KN, van de Graaf SF. Homo- and heterodimeric architecture of the human liver Na^+-dependent taurocholate cotransporting protein NTCP. Biochem J 2012 Feb 1;441(3):1007-15.
2. Annaert PP, Brouwer KL. Assessment of drug interactions in hepatobiliary transport using rhodamine 123 in sandwich-cultured rat hepatocytes. Drug Metab Dispos 2005;33:388-394.
3. Oude Elferink RP, Ottenhoff R, van Wijland M, Smit JJ, Schinkel AH, Groen AK. Regulation of biliary lipid secretion by mdr2 P-glycoprotein in the mouse. J Clin Invest 1995;95:31-38.
4. Schieck A, Müller T, Schulze A, Haberkorn U, Urban S, Mier W. Solid-phase synthesis of the lipopeptide Myr-HBVpreS/2-78, a hepatitis B virus entry inhibitor. Molecules 2010;15:4773-4783.

5. Mier W, Hoffend J, Kramer S, Schuhmacher J, Hull WE, Eisenhut M, Haberkorn U. Conjugation of DOTA using isolated phenolic active esters: the labeling and biodistribution of albumin as blood pool marker. Bioconjug Chem 2005;16:237-240.

Supplementary tables and figures

Supp. table 1: Primer sequences used for amplification of the mouse *Slc10a1* gene by polymerase chain reaction (PCR).

Gene name	Sense and antisense	Product length
Slc10a1 (wild-type allele)	5'-CATCTGACCAGCATTGAGGC-3'	412-bp
	5'-GTTCTGAGGAACTCTTTCATC-3'	
Slc10a1 (targeted allele)	5'-GGTCTCCGAGACTGAGCTAG-3'	624-bp
	5'-CCCTAGGAATGCTCGTCAAGA-3'	

Supp. table 2: Oligonucleotide primers used for qRT-PCR analysis of wild-type and *Slc10a1*$^{-/-}$ (each used at a final concentration of 5 µM). Genes of interest are involved in hepatic sinusoidal BA uptake (*Slc10a1, Slco1a1, Slco1a4, Slco1b2*), intestinal and renal BA transport (*Slc10a2, Slc51a and Abcc2*). Reference genes (*Rplp0, Tbp, Ppib and Hprt*) are also shown.

Gene name	Sense and antisense	Product length	Used in
Slc10a1	5'-TGGCTACCTCCTCCCTGATG-3'	380-bp	Liver
	5'-GCCAGGTTGTGTAGGAGGAT-3'		
Slco1a1	5'-TGAGAAAGACAGCAGTAGGACTTT-3'	162-bp	Liver
	5'-GTGATTTGGCTAGGTATGCAC-3'		
Slco1a4	5'-TACATGTCAGCTTGCCTCGC-3'	140-bp	Liver
	5'-GCACACTCAGGACCCTTGTC-3'		
Slco1b2	5'-GGGTGAATGCCCAAGAGACA-3'	282-bp	Liver
	5'-TATAGCCTGCATGCTCCACG-3'		
Slc10a2	5'-CCATGGGGTATCTTCGTGGG-3'	278-bp	Ileum, kidney
	5'-GTTCCCGAGTCAACCCACAT-3'		
Slc51a	5'-GGCATCTATGACCCAGGAGA-3'	151-bp	Ileum, kidney
	5'-TGGATCCCATGTTCTGTTCA-3'		
Slc51b	5'-GACCACAGTGCAGAGAAAGC-3'	102-bp	Liver
	5'-ATTCCAAGGAGCCGCATCT-3'		
Abcc2	5'-AGCAGGTGTTCGTTGTGTGT-3'	133-bp	Kidney
	5'-AGCCAAGTGCATAGGTAGAGAAT-3'		
Rplp0	5'-CCAGCGAGGCCACACTGCTG-3'	169-bp	Liver, kidney
	5'-ACACTGGCCACGTTGCGGAC-3'		
Tbp	5'-GGAGAATCATGGACCAGAACA-3'	89-bp	Liver
	5'-GATGGGAATTCCAGGAGTCA-3'		

Ppib	5'-TCGGAGCGCAATATGAAGGT-3'	65-bp	Ileum
	5'-AAAAGGAAGACGACGGAGCC-3'		
Hprt	5'-TTGCTCGAGATGTCATGAAGGA-3'	91-bp	Ileum
	5'-AGCAGGTCAGCAAAGAACTTATAG-3'		

Supp. table 3: Liver weights and biochemical indicators in serum of 2-month-old wild-type, normocholanemic and hypercholanemic *Slc10a1*$^{-/-}$ mice. Female mice were included (n=4-5 per group). AST: aspartate aminotransferase. ALT: alanine aminotransferase. ALP: alkaline phosphatase. Values are given as mean ± S.E.M. Asterisk indicates p-value below 0.05.

Parameter	Unit	wild-type	KO low BA	KO high BA
Liver weight	% of body weight	5.2 ± 0.2	5.0 ± 0.2	5.2 ± 0.2
Serum total bilirubin	µmol/L	3.2 ± 0.7	7.2 ± 3.4	24.8 ± 7.5 *
Serum conjugated bilirubin	µmol/L	2.6 ± 0.6	8.2 ± 5.8	31.4 ± 8.0 *
Serum AST	U/L	142.5 ± 33.1	186.7 ± 43.6	896.4 ± 403.3
Serum ALT	U/L	58.0 ± 12.5	51.0 ± 15.8	152.8 ± 47.6
Serum ALP	U/L	22.0 ± 6.2	15.9 ± 4.6	219.1 ± 38.8 *

Supp. figure 1: (A) NTCP protein (~45-50 kDa) levels in liver membranes of wild-type (WT) and heterozygous (HET) *Slc10a1* mice. Values are given as mean ± S.E.M., quantified relative to sodium/potassium ATPase (~ 90 kDa) and normalized to wild-type levels (set at 1.0). Liver membrane homogenate of one *Slc10a1*$^{-/-}$ (KO) mouse was included as negative control. (B) Mouse NTCP protein size without (-) and with (+) *N*-glycosidase PNGase F treatment (left panel). As a positive control, deglycosylation of HA-tagged hNTCP, stably transfected in U2OS cells, was shown by Western blot analysis (right panel). β-actin served as a loading control.

Supp. figure 2: Liver hematoxylin-eosin histology comparing adult male wild-type and *Slc10a1*$^{-/-}$ mice. Both knockout mice with normal serum BAs and high serum BAs were investigated. Section thickness is 7 µm. Portal vein (pv) and central vein (cv) are indicated. Scale bar is 100 µm.

Supp. figure 3: Relative mRNA expression levels of the *Oatp*-isoforms were calculated for wild-type and hypercholanemic *Slc10a1*$^{-/-}$ mice upon dietary supplementation with 0.1% UDCA. Values are given as mean ± S.E.M. for 4-6 adult female mice per group. Asterisk indicates p-value below 0.05.

Supp. figure 4: (A) Schematic representation of ^{68}Ga-WT peptide (top) and ^{68}Ga-control peptide (bottom). (B) microPET-based pretesting of the Myrcludex-B-derived peptides in female wild-type NMRI mice. Top: Uncorrected planar projection, bottom: coronal PET section.
